# Evidence against Stable Protein S-Nitrosylation as a Widespread Mechanism of Post-translational Regulation

**DOI:** 10.1016/j.molcel.2017.12.019

**Published:** 2018-02-01

**Authors:** Kathryn Wolhuter, Harry J. Whitwell, Christopher H. Switzer, Joseph R. Burgoyne, John F. Timms, Philip Eaton

**Affiliations:** 1 King’s College London, School of Cardiovascular Medicine & Sciences, British Heart Foundation Centre of Research Excellence the Rayne Institute, St. Thomas’ Hospital, London SE1 7EH, UK; 2 Institute for Women’s Health, University College London, Gower Street, London WC1E 6BT, UK

**Keywords:** S-nitrosation, S-nitrosylation, disulfide, protein, cysteine, thiol, redox, signaling

## Abstract

S-nitrosation, commonly referred to as S-nitrosylation, is widely regarded as a ubiquitous, stable post-translational modification that directly regulates many proteins. Such a widespread role would appear to be incompatible with the inherent lability of the S-nitroso bond, especially its propensity to rapidly react with thiols to generate disulfide bonds. As anticipated, we observed robust and widespread protein S-nitrosation after exposing cells to nitrosocysteine or lipopolysaccharide. Proteins detected using the ascorbate-dependent biotin switch method are typically interpreted to be directly regulated by S-nitrosation. However, these S-nitrosated proteins are shown to predominantly comprise transient intermediates leading to disulfide bond formation. These disulfides are likely to be the dominant end effectors resulting from elevations in nitrosating cellular nitric oxide species. We propose that S-nitrosation primarily serves as a transient intermediate leading to disulfide formation. Overall, we conclude that the current widely held perception that stable S-nitrosation directly regulates the function of many proteins is significantly incorrect.

## Highlights

• Protein S-nitrosation is commonly regarded as a stable, regulatory modification
• However, S-nitrosothiols are labile and rapidly react with thiols to form disulfides
• Here disulfides are shown to be the dominant end effectors of nitrosative signaling
• Protein S-nitrosation as a regulatory end effector may occur, but this may be rare

## Graphical Abstract

**Figure:**
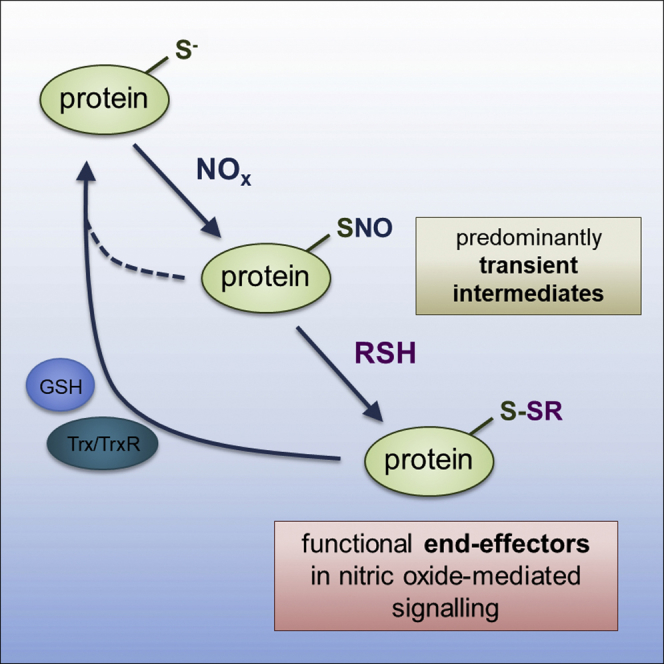

## Teaser

Nitric oxide-related molecules post-translationally modify protein thiols, resulting in functional changes. Such S-nitrosothiols are widely thought to be regulatory end effector modifications. However, Wolhuter et al. show that S-nitrosation predominantly serves as a transient intermediate in the formation of disulfide bonds. This furthers our understanding of how nitric oxide regulates signaling.

## Introduction

Protein S-nitrosation, often referred to as S-nitrosylation, is a post-translational modification involving the covalent addition of a nitrosonium (NO^+^) equivalent to the sulfur atom of cysteine. The mechanisms by which endogenous S-nitrosothiols are formed are unclear. Nitrosothiol formation may occur via oxidation of NO to N_2_O_3_ in the presences of an electron acceptor (Broniowska and Hogg, 2012) or radical recombination between a thiyl radical and NO (Madej et al., 2008). Alternatively, dinitrosyl-iron or heme-NO pathways may be involved (Bosworth et al., 2009, Jia et al., 1996), although the physiological occurrence of such reactions remains unclear (Gladwin et al., 2003). Protein S-nitrosation is widely held as a principal mechanism by which changes in cellular NO are transduced to exert its biological effects. The biological significance of S-nitrosation is supported by studies showing that nitrosothiol formation at the active site of proteins inhibits their activity (Mannick et al., 2001).

S-nitrosation is promoted as an evolutionarily conserved signaling mechanism that affects most, if not all, classes of proteins. Direct parallels have been drawn between S-nitrosation and established regulatory end effector modifications, such as phosphorylation (Hess and Stamler, 2012). For example, NO synthase enzymes co-locate with target proteins (Dejanovic and Schwarz, 2014), operating as “nitrosylases” in an analogous role to kinases mediating phosphorylation. S-nitrosation is reported to be selective by preferentially targeting particular amino acid consensus sequence motifs (Stamler et al., 1997), again mirroring a feature of regulation by kinases. Similarly “denitrosylase” enzymes that remove NO from proteins, the equivalent of phosphatases, have been reported (Benhar et al., 2009). Regulatory protein S-nitrosation is also supported by work showing that it can occur stoichiometrically (Mannick et al., 2001), that genetic ablation of a target cysteine can abrogate the functional effect of a nitrosating intervention (Leiper et al., 2002), and that pharmacological inhibition of a denitrosylase enzyme promotes S-nitrosation (Cole et al., 2009). Finally, genetic knockout of an NO synthase enzyme *in vivo* decreases protein S-nitrosation (Burger et al., 2009). Consequently, it would appear irrefutable that stable protein S-nitrosation is a widespread regulatory mechanism. Despite this widely held view, in this study, we present experimental evidence and logical arguments challenging this ideology. S-nitrosated thiols are intrinsically unstable, especially in the intracellular context, where they react with reduced metal ions as well as abundant thiols to generate disulfides (Arnelle and Stamler, 1995). Such disulfides are well-established post-translational modifications with greater stability than nitrosated thiols, long known for their regulatory roles (Rudyk et al., 2013). Because the cellular environment is highly reducing, abundant in glutathione (GSH) and other thiols, the lifetime of most S-nitrosated thiols is likely to be only transient (Singh et al., 1996).

With the instability of the protein S-nitrosothiol in mind, as well as other considerations presented in the Discussion, experiments were performed to investigate the rational hypothesis that protein S-nitrosation is predominantly a transient intermediate leading to disulfide formation. This work is not intended to challenge the existence of S-nitrosothiols or that they serve important roles in biological systems. Instead, evidence is presented that challenges the widely held paradigm that stable S-nitrosation is a ubiquitous post-translational modification that directly regulates proteins function. Furthermore, this does not mean that stable protein S-nitrosation cannot occur but, rather, that the extent to which it widely serves as an end effector modification is likely overestimated.

## Results

### Disulfide Bond Formation Precedes an Increase in Observable Protein S-Nitrosothiols

**Figure 1:**
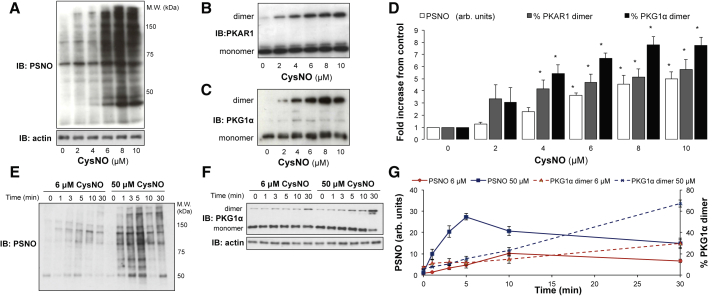
Disulfide Formation Is Observed before an Increase in Detectable S-Nitrosothiols (A–C) SMCs treated with increasing doses of CysNO for 30 min were analyzed for protein S-nitrosothiol (PSNO) content using the biotin switch method. A dose-dependent increase in PSNO (A) was observed as well as a concurrent increase in PKAR1 (B) and PKG1α (C) disulfide dimer. (D) A significant increase in disulfide dimer formation in both proteins was observed before a significant change in detectable PSNO. (E and F) PSNO and PKG1α formation measured by immunoblotting over a time course of 30 min after SMCs were treated with 6 or 50 μM CysNO. (G) PSNO peaked at 10 and 5 min, respectively, before decreasing in abundance, whereas the PKG1α disulfide dimer increased with time. Data are represented as mean ± SEM; ^∗^p < 0.05.

Exposure of rat aortic smooth muscle cells (SMCs) to 0–10 μM S-nitrosocysteine (CysNO) caused a dose-dependent increase in detectable S-nitrosothiols (Figure 1A), reaching statistical significance at 6 μM (Figure 1D). A concomitant increase in disulfide dimerized protein kinase A regulatory subunit (PKAR) 1 and protein kinase G (PKG) 1α was also observed (Figures 1B and 1C). It was especially notable that a significant increase in PKAR1 and PKG1α disulfide levels preceded the increase in detectable, stable S-nitrosothiols (Figure 1D; p < 0.05). S-nitrosothiol and PKG1α disulfide formation in SMCs was measured over a time course of 30 min after the addition of 6 or 50 μM CysNO (Figures 1E and 1F). Detectable S-nitrosothiols peaked at 10 or 5 min, respectively, before decreasing in abundance, whereas PKG1α disulfide dimerization continuously increased over the 30 min (Figure 1G). These data are consistent with the formation of transient S-nitrosothiols that rapidly transition to disulfides.

**Figure 2:**
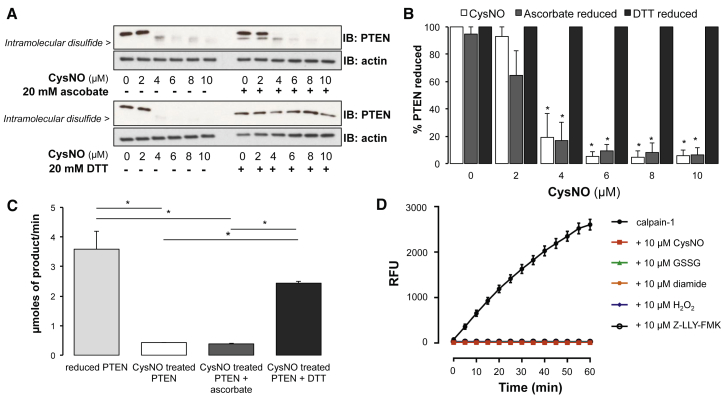
Functional Changes Regulated by Disulfides, Not S-Nitrosation (A) The oxidation state of PTEN was measured in SMCs treated with CysNO. (B) After exposure of CysNO, there was a decrease in reduced PTEN. Reduced PTEN was observed after reduction by DTT but not ascorbate. (C) PTEN’s enzymatic activity was inhibited after exposure to CysNO, as measured by *in vitro* phosphatase activity assay. Enzyme activity was restored after addition of DTT but not ascorbate. (D) Calpain-1’s enzymatic activity was inhibited *in vitro* after exposure to the nitrosating agent CysNO, the disulfide-inducing reagents GSSG, H_2_O_2_, or diamide, or the irreversible calpain inhibitor Z-LLY-FMK. Data are represented as mean ± SEM; ^∗^p < 0.05.

CysNO induced oxidation of phosphatase and tensin homolog (PTEN), a protein known to form an intraprotein disulfide during oxidant stress (Lee et al., 2002). Formation of intraprotein disulfide between Cys71–Cys124 can be detected using anti-PTEN antibody. Unfortunately, disulfide formation in PTEN after treatment with CysNO interfered with binding of the detection antibody (Figure 2A), likely because of oxidation other than the Cys71–Cys124 intraprotein disulfide interfering with the antibody’s epitope. Consequently, oxidation was indexed as loss of reduced PTEN (Figure 2B), which was dose-dependent and again preceded detection of protein S-nitrosation. Reduction with the nitrosothiol reductant ascorbate did not reduce PTEN, whereas chemical reduction with DTT restored PTEN to its reduced state (Figure 2B; p < 0.05). This indicates that CysNO induced disulfide bond formation in PTEN but not stable S-nitrosation. PTEN enzyme activity was significantly inhibited after exposure to CysNO, as measured using an *in vitro* phosphatase activity assay. Reduction with the nitrosothiol reductant ascorbate did not restore activity of the enzyme, whereas the disulfide reductant DTT recovered activity to that of untreated PTEN (Figure 2C; p < 0.05). This indicates that inhibition of PTEN is mediated by a disulfide and not by stable S-nitrosation.

The cysteine protease calpain-1 is reportedly directly inhibited by S-nitrosation of its active-site thiol (Liu et al., 2016). Although the nitrosating agent CysNO inhibited calpain-1 activity, as measured using an *in vitro* calpain activity assay, comparable inhibition was achieved by the disulfide-inducing reagents glutathione disulfide (GSSG) diamide, or H_2_O_2_ (Figure 2D). These data are consistent with caplain-1 inhibition via disulfide formation.

### Multiple NO Donors Induce Protein S-Thiolation

**Figure 3:**
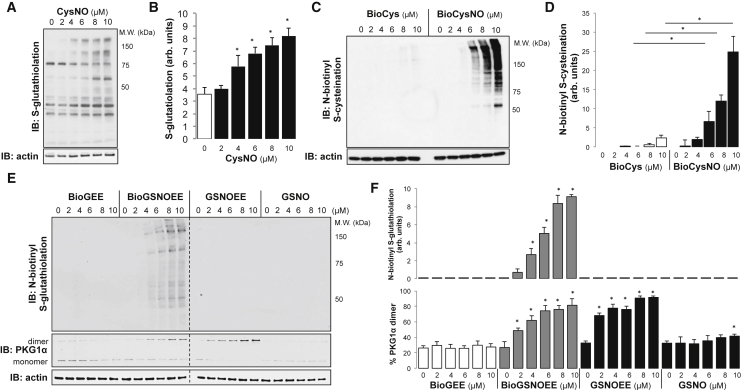
An NO Donor Induces S-Thiolation (A) SMCs were exposed to CysNO for 30 min, and a dose-dependent increase in S-glutathiolated proteins was detected by immunoblotting. (B) A significant increase in S-glutathiolated proteins was observed at concentrations of CysNO above 4 μM. (C and D) A significant increase in S-cysteinated proteins was observed when SMCs were treated with biotin-CysNO (BioCysNO) compared with the biotin-cysteine (BioCys) control. (E) SMCs were treated with N-biotinyl glutathione ethyl ester (BioGEE), N-biotinyl S-nitrosoglutathione (GSNO) ethyl ester (BioGSNOEE), S-nitrosoglutathione ethyl ester (GSNOEE), or GSNO. N-biotinyl S-glutathiolated proteins and PKG1α were detected by immunoblotting. (F) BioGSNOEE induced significant S-glutathiolation compared with that induced by the non-nitrosated BioGEE control. BioGSNOEE or GSNOEE both also induced significant disulfide dimerization of PKG1α. Data are represented as mean ± SEM; ^∗^p < 0.05.

S-thiolation refers to disulfide bond formation between a protein thiol and a non-protein thiol. CysNO exposure resulted in dose-dependent S-glutathiolation in SMCs (Figures 3A and 3B; p < 0.05). As with the other disulfide modifications monitored (Figures 1D and 2B), significant increases in protein S-glutathiolation preceded an accumulation in detectable S-nitrosothiols (Figure 3B). To monitor protein S-cysteination, biotin-cysteine (BioCys) was used to generate N-biotinyl-S-nitrosocysteine (BioCysNO). Protein S-cysteination significantly increased after SMC exposure to 6 μM BioCysNO compared with 6 μM BioCys alone (Figures 3C and 3D; p < 0.05), consistent with CysNO inducing significant S-thiolation in SMCs.

Protein S-glutathiolation induced by the NO donor S-nitrosocysteine (GSNO) was determined using an N-biotinylated, cell-permeable derivative of GSNO. SMCs were treated with 0–10 μM N-biotinyl GSNO ethyl ester (BioGSNOEE) as well as the control reagents N-biotinyl glutathione ethyl ester (BioGEE), GSNO ethyl ester (GSNOEE) or GSNO. N-biotinyl S-glutathiolation and disulfide dimer formation in PKG1α were measured in all samples (Figure 3E). Protein S-glutathiolation was significantly increased by treatment with BioGSNOEE but not BioGEE (Figure 3F; p < 0.05). Significant disulfide dimerization of PKG1α was induced by 2 μM BioGSNOEE or GSNOEE, each of which are cell-permeable. Cell-impermeable GSNO induced a small but significant increase in PKG1α disulfide formation at a higher concentration of 10 μM, whereas BioGEE did not alter its redox state (Figure 3F; p < 0.05). These data are consistent with GSNO inducing PKG1α dimer formation and S-glutathiolation via transient S-nitrosothiol intermediates.

### Detection of S-Nitrosothiols Dependent on Cellular Glutathione Levels

**Figure 4:**
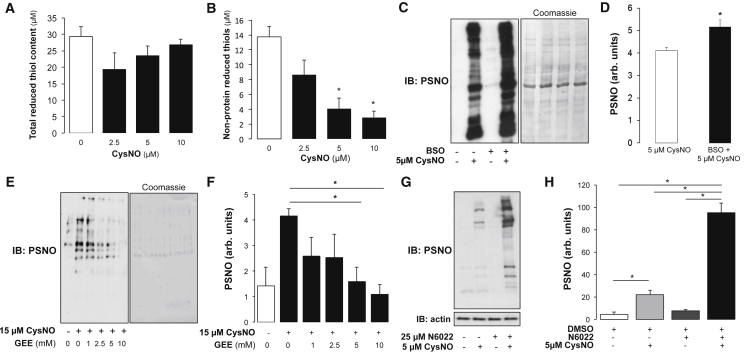
Detection of S-Nitrosothiols Is Dependent on the Abundance of GSH (A and B) Ellman’s assay was performed to detect total and non-protein thiols in SMCs exposed to CysNO for 30 min. Although there was no change in total thiol concentration (A), a significant decrease in non-protein thiols was observed with increasing CysNO concentration (B). (C) Glutathione synthesis was inhibited in SMCs for 24 hr using buthionine sulfoximine (BSO) to deplete the cellular GSH concentration. SMCs exposed to CysNO were analyzed for PSNO content using the biotin switch method. (D) Depletion of GSH significantly increases detectable PSNO. (E) SMCs were exposed to CysNO, followed by treatment with increasing doses of GSH ethyl ester (GEE) to augment cellular GSH concentration. (F) A dose-dependent decrease in PSNO was detected after treatment with GEE, reaching significance at 5 mM GEE. (G) SMCs were pre-treated with or without the S-nitrosoglutathione reductase inhibitor N6022 for 1 hr prior to exposure to CysNO. (H) Exposure to N6022 significantly potentiated the accumulation CysNO-induced PSNO. Data are represented as mean ± SEM; ^∗^p < 0.05.

Exposure of SMCs to CysNO did not significantly alter total reduced thiols but dose-dependently depleted non-protein reduced thiols, comprised predominantly of GSH (Figures 4A and 4B; p < 0.05). Pharmacological depletion of cellular GSH using buthionine sulfoximine prior to CysNO treatment significantly increased protein S-nitrosation (Figures 4C and 4D; p < 0.05), consistent with an impaired ability to reduce the nitrosothiols via S-glutathiolation. Conversely, pharmacologically augmenting cellular GSH after CysNO treatment by supplying cell-permeable reduced GSH ethyl ester significantly attenuated protein S-nitrosation (Figures 4E and 4F; p < 0.05). Although treatment of SMCs with the selective S-nitrosoglutathione reductase (GSNOR) inhibitor N6022 alone did not increase the detection of S-nitrosothiols, inhibition of GSNOR significantly potentiated S-nitrosation after exposure to CysNO (Figures 4G and 4H; p < 0.05). These findings are consistent with S-nitrosated proteins accumulating because the reducing capacity of the cell was compromised, with re-supply of reduced GSH leading to rapid denitrosation. These findings illustrate that interventions that compromise the cellular reducing status permit S-nitrosothiol levels that otherwise may not occur.

### S-Nitrosating Agents Compromise the Ability of Cells to Recycle Disulfides

**Figure 5:**
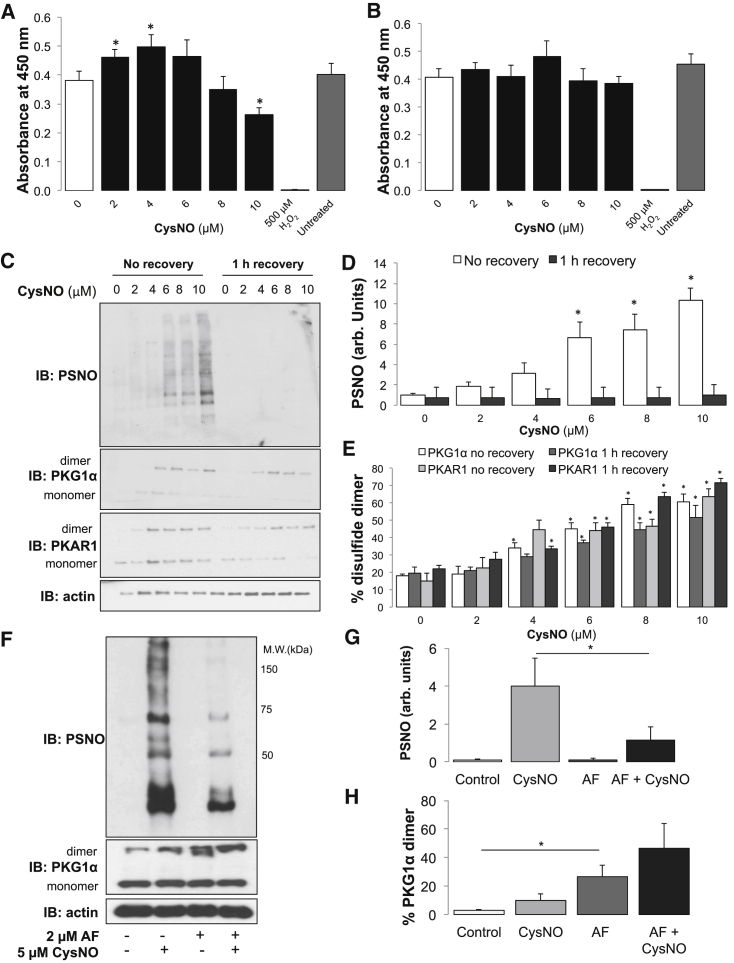
The NO Donor Reduces SMCs’ Capacity to Recycle Disulfides (A) The activity of NADPH-dependent enzymes was measured using the 3-(4,5-dimethylthiazol-2-yl)-5-(3-carboxymethoxyphenyl)-2-(4-sulfophenyl)-2H-tetrazolium (MTS) assay after SMCs were exposed to CysNO. Enzymes were significantly inhibited after exposure to 10 μM CysNO for 30 min. (B) When SMCs were allowed to recover for 1 hr after exposure to CysNO, changes in activity were abolished. (C) PSNOs were detected using the biotin switch method in SMCs that had no recovery or 1 hr recovery after CysNO treatment. SMCs that had no recovery period showed a dose-dependent increase in PSNO along with a concurrent increase in PKAR1 and PKG1α disulfide dimers. After a recovery period of 1 hr, there was no observable increase in PSNO, but a dose-dependent increase in PKAR1 and PKG1α disulfide dimers was apparent. (D) A recovery period of 1 hr abolished the detection of PSNO following treatment with CysNO. (E) A significant increases in PKAR1 and PKG1α disulfide dimers were observed in both SMCs with no recovery and after 1 hr recovery. (F) SMCs were treated with AF for 1 hr prior to exposure to CysNO, and then PSNO, PKG1α and α-actin were measured. (G) Treatment with AF prior to CysNO exposure significantly decreased observable PSNO. (H) AF treatment induced a significant increase in the PKG1α disulfide dimer that was exacerbated with exposure to CysNO. Data are represented as mean ± SEM; ^∗^p < 0.05.

Nicotinamide adenine dinucleotide phosphate (NADPH)-dependent enzymes mediate the recycling of cellular disulfides back to their reduced, free thiol state. Although 2–4 μM CysNO was shown to increase the activity of NADPH-dependent enzymes slightly, exposure of SMCs to 10 μM CysNO significantly decreased their activity, as indexed by a tetrazolium dye reduction assay. In contrast, when SMCs were allowed to recover for 60 min after CysNO exposure, NADPH-dependent activity returned to basal levels (Figures 5A and 5B; p < 0.05). This is consistent with CysNO reversibly inhibiting NADPH-dependent enzymes that include Trx reductase (TrxR) and GSH reductase. Incubation of SMCs with CysNO elevated both S-nitrosothiols and disulfide PKAR1 and PKG1α levels as before. Following 1 hr of recovery, S-nitrosothiols levels returned to basal, whereas the kinase disulfides remained elevated (Figures 5C–5E). This indicates that, when the reducing activity of the cell is restored, S-nitrosothiols are unstable compared with the disulfides monitored. Pharmacological inhibition of TrxR by pre-treatment with auranofin (AF) significantly reduced SMCs protein S-nitrosation induced by CysNO. In contrast, AF increased PKG1α disulfide dimer levels and potentiated oxidation of the kinase by CysNO (Figures 5F–5H; p < 0.05). These observations are consistent with AF inhibiting the disulfide recycling system, compromising the availability of reduced thiols that otherwise reduce S-nitrosated proteins to yield disulfides.

### Disulfides Are the Dominant Product of Exogenous or Endogenous Nitrosative Signaling

**Figure 6:**
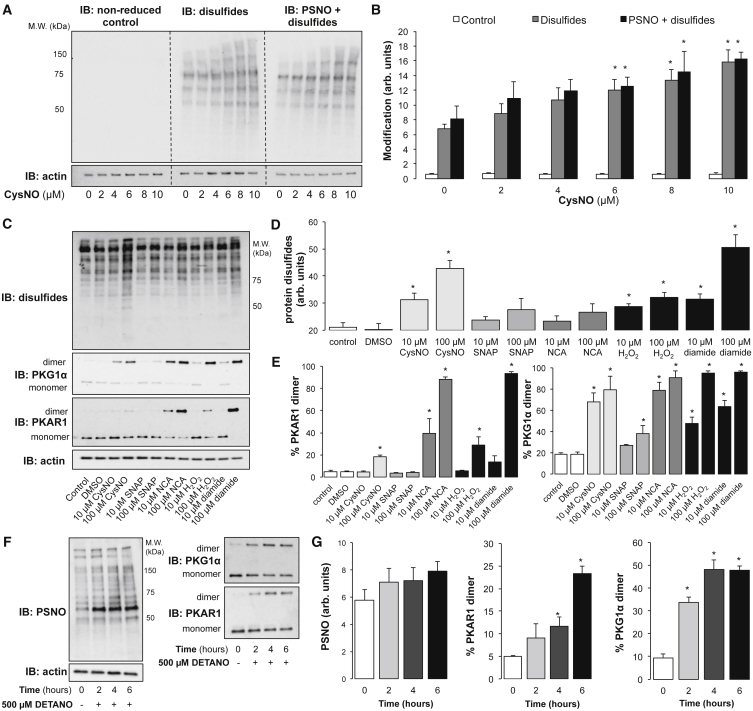
The Major Product of Exogenous or Endogenous Nitrosative Signaling Is Disulfide Formation (A) The double S-nitrosothiol/disulfide switch was used to compare disulfides with combined (PSNO + disulfides) protein oxidation in SMCs after treatment with CysNO for 30 min. A dose-dependent increase in total protein oxidation was observed upon treatment with CysNO. (B) A significant increases in both disulfides and PSNO + disulfides was observed from baseline. There was no statistical difference between the two treatment groups at any concentration of CysNO. (C) Global disulfides and disulfide formation in PKG1α and PKAR1 was measured in SMCs after 5 min treatment with 10 or 100 μM CysNO, SNAP, NCA, H_2_O_2_, or diamide. (D) Global disulfides were significantly increased after exposure to both doses of CysNO, H_2_O_2_, or diamide. (E) Significant disulfide formation in PKAR1 was induced by 10 μM NCA or 100 μM CysNO, H_2_O_2_, or diamide. Significant PKG1α dimerization occurred after exposure to 10 or 100 μM CysNO, NCA, H_2_O_2_, or diamide or 100 μM SNAP. (F) PSNO, PKG1α, and PKAR1 disulfide dimer formation was measured in SMCs over a 6-hr treatment with 500 μM DETANO. (G) Although no significant increase in PSNO was observed at any time point, a significant increase in PKAR1 disulfide formation was observed after 4 hr and PKG1α at 2 hr. Data are represented as mean ± SEM; ^∗^p < 0.05.

It is evident that exogenous NO donors induce widespread protein S-nitrosation together with concomitant global disulfide formation. Such observations are consistent with transient S-nitrosothiol intermediates leading to disulfides but do not preclude the possibility that many proteins do not transition to disulfides and were present as undetected, stable S-nitrosothiols. To address this possibility, we used an approach that enabled the simultaneous, direct quantitative comparison of protein S-nitrosation versus disulfide, allowing us to determine the extent to which multiple protein thiols are occupied by NO versus a disulfide after a nitrosative intervention. Consequently, we performed a double S-nitrosothiol/disulfide switch utilizing selective chemical reduction and labeling steps to allow the degree of protein cysteine oxidation because of disulfides to be compared with that arising from a combination of S-nitrosation and disulfide (Figure 6A). A significant increase in total protein oxidation signal (i.e., combined S-nitrosation and disulfide) was observed at 6 μM CysNO (Figure 6B; p < 0.05). When comparing signals generated by disulfides alone with S-nitrosothiols and disulfides combined, there was no significant difference (Figure 6B; p < 0.05). This indicates that disulfides account for the vast majority of the signal, with only a small proportion arising from stable S-nitrosothiols. Thus, the predominant outcome of exposing SMCs to CysNO is disulfide formation, although, clearly, robust protein S-nitrosation concomitantly occurs.

To determine the efficiency with which nitrosating agents induce disulfide formation compared with other reactive nitrogen/oxygen species, global and candidate protein disulfides were measured in SMCs in response to 5 min of low (10 μM) or high (100 μM) doses of various oxidants. SMCs were treated with the cell-permeable transnitrosating agent CysNO, the cell-impermeable transnitrosating agent S-nitroso-N-acetyl-DL-penicillamin (SNAP), the nitroxyl donor 1-nitrosocyclohexyl acetate (NCA), H_2_O_2_, or diamide, with global disulfides indexed using a disulfide switch method (Figure 6C). Significant increases in global disulfides were observed after exposure to either dose of CysNO, H_2_O_2_, or diamide, with the efficiency of CysNO comparable with the thiol-oxidizing agent diamide (Figure 6D; p < 0.05). Disulfide formation in PKAR1 was significantly increased after exposure to either dose of NCA or a high dose of CysNO, H_2_O_2_, or diamide (Figure 6E; p < 0.05). Significant disulfide PKG1α was observed after exposure to either dose of CysNO, NCA, H_2_O_2_, or diamide or the high dose of SNAP (Figure 6E; p < 0.05). These data highlight the propensity of nitrosating agents to efficiently induce disulfide formation. In addition, although NCA was highly effective at inducing disulfide formation in PKAR1 and PKG1α, it did not significantly increase global disulfides. This indicated that CysNO-induced increases in global protein disulfides were not likely via CysNO-dependent nitroxyl formation.

To establish whether elevations in authentic NO mirrored observations made when using transnitrosating NO donors, protein S-nitrosothiols and candidate protein disulfides were measured in SMCs after treatment with diethylenetriamine/nitric oxide (DETANO), a slow-releasing NO compound, over 6 hr (Figure 6F). In support of previous observations, although there was no significant increase in S-nitrosation at any time point, disulfide dimerization in PKAR1 and PKG1α was significantly increased at 4 or 2 hr, respectively (Figure 6G; p < 0.05).

**Figure 7:**
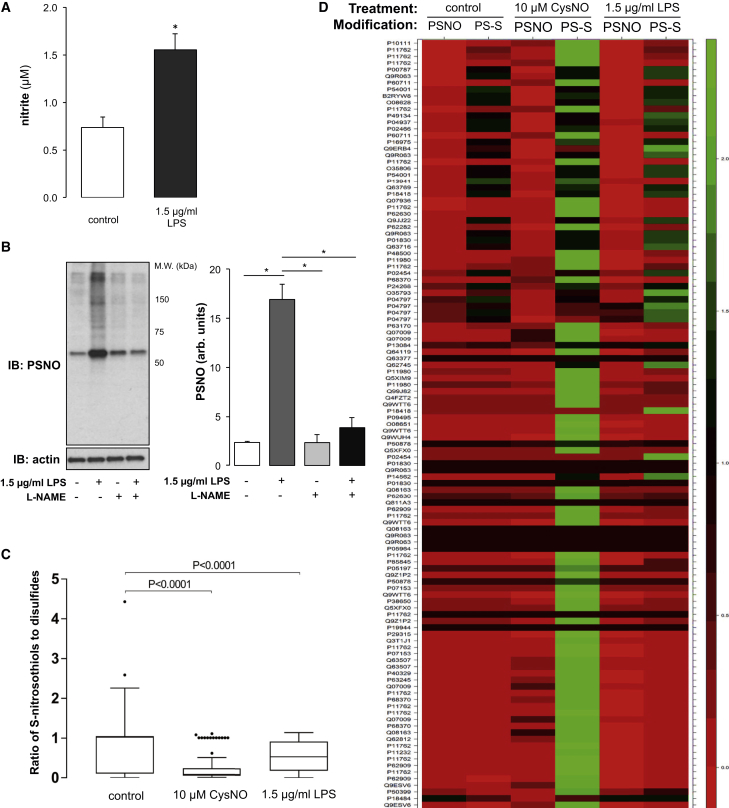
Protein Thiols that Form S-Nitrosothiols Preferentially Form Disulfides (A) Using the Griess assay, an increase in nitrite was detected in SMCs treated with LPS for 18 hr, signifying elevated NO production. (B) Increased NO production correlated with a small but significant increase in PSNO. Increased PSNO was attenuated after treatment with the pan-specific NOS inhibitor L-NAME. Data are represented as mean ± SEM; ^∗^p < 0.05. (C) PSNO and disulfides were labeled with iodoTMT sixplex reagents in SMCs treated with 10 μM CysNO, 1.5 μg/mL LPS, or under control conditions. Analysis by LC-MS/MS showed that the ratio of S-nitrosothiol to disulfide significantly decreased after SMCs were exposed to exogenous or endogenous nitrosative signaling. (D) The heatmap depicts the scaled and centered peptide peak area of iodoTMT-labeled PSNO or disulfide (PS-S) peptides under each condition. Accession numbers indicate the modification of an individual cysteine residue within a given protein. The color tracker represents the *Z* score for each cysteine modification and ranges from 0 (red), representing low-abundance modifications, to 2 (green), representing high-abundance modifications.

To expand on these observations, and in an attempt to detect stable S-nitrosated protein formation, an unbiased, multiplexed proteomics approach was used. This combined iodoacetyl tandem mass tags (iodoTMTs) with selective reductive labeling of S-nitrosothiols or disulfides, allowing the quantitative comparison of each of these nitro-oxidative modifications in SMCs after exogenous or endogenous nitrosative signaling. Stimulation of SMCs with 1.5 μg/mL lipopolysaccharide (LPS) for 18 hr increased endogenous NO, indicated by a 2-fold increase in nitrite as measured by the Griess assay (Figure 7A; p < 0.05). Increased NO production correlated with a small but significant increase in S-nitrosothiols, as detected by the ascorbate-dependent biotin switch (Figure 7B; p < 0.05). Accumulation of S-nitrosothiols was abolished when cells were co-treated with the nitric oxide synthase inhibitor L-N^G^-Nitroarginine methyl ester (L-NAME), indicating that the increase in protein S-nitrosation was via activation of nitric oxide synthase (Figure 7B; p < 0.05). S-nitrosothiols and disulfides present in SMCs basally and in cells treated with 10 μM CysNO for 30 min or 1.5 μg/mL LPS for 18 hr were detected using selective reduction and labeling with iodoTMT sixplex reagents. The three samples were then combined, fractionated, and analyzed using liquid chromatography-tandem mass spectrometry. Mass spectrometry analysis allowed the detection of basal, low-abundance S-nitrosothiol or disulfide-containing proteins, identifying 118 labeled cysteine sites from 68 proteins that formed S-nitrosothiols or disulfides or underwent both modes of oxidation (Figures 7C and 7D). After exogenous or endogenous nitrosative signaling, S-nitrosothiols as well as disulfides increased reproducibly. The ratio of S-nitrosothiol to disulfide was calculated using the intensity of iodoTMT-labeled peptides for each cysteine under control conditions and after treatment with CysNO or LPS. Under control conditions, a ratio of 1:1 represents the detection of very-low-abundance modifications with similar stoichiometry (Figure 7C). After SMCs were exposed to CysNO or LPS, the detection of both S-nitrosothiols and disulfides was increased; however, the overall ratio of S-nitrosothiols to disulfides significantly decreased, consistent with disulfides, not S-nitrosothiols, being the dominant modification resulting from either of the nitrosative signaling interventions (Figure 7C).

The intensity of iodoTMT-labeled S-nitrosothiol or disulfide peptides under the three treatment conditions was plotted as a heatmap, with accession numbers relating to specific cysteines located within that protein (Figure 7D). This unbiased proteomics analysis showed that SMCs exposed to 10 μM CysNO or 1.5 μg/mL LPS increased protein S-nitrosation. However, this increased S-nitrosation was quantitatively very minor compared with the corresponding increase in protein disulfide formation at the same cysteine sites. The heatmap highlights that, in the majority of cases, cysteines that form S-nitrosothiols also formed disulfide bonds and, crucially, that these disulfides occurred with higher stoichiometry than S-nitrosothiols (Figure 7D). These data indicate that an overwhelming majority of protein thiols that become S-nitrosated are also present in the disulfide state, which is clearly the dominant modification in terms of stoichiometry or target occupancy. Endogenous or exogenous nitrosative signaling induces widespread protein S-nitrosation, but, in most cases, these are not stable and so do not accumulate stoichiometrically, consistent with them transitioning to disulfides, which are prevalent.

## Discussion

The principal findings of this study suggest that protein S-nitrosation, in most cases, is primarily a short-lived, intermediate in the formation of disulfide bonds. The formation of these interprotein, intraprotein, or S-thiolation disulfides is shown to be the predominant post-translational modification in a wide range of proteins following exogenous and endogenous interventions that promote protein S-nitrosation.

As expected, when cells were exposed to CysNO and then analyzed by the biotin switch method, strong and widespread increases in protein S-nitrosation occurred, as previously reported. These signals are robust, so it is instinctive to interpret them as evidence of cell-wide stable protein S-nitrosation. The next seemingly logical inference, having witnessed such robust post-translational modification signals, is that these represent proteins directly regulated by S-nitrosation. However, this conclusion ignores the fact that the stability and stoichiometry of the modification is unknown.

Although S-nitrosothiols are typically represented in the literature as stable, regulatory modifications, it is recognized that they efficiently react with thiols to generate disulfides (Broniowska and Hogg, 2012). Under the prevalent reducing conditions of the cytosol, where thiol-containing molecules are present in millimolar concentrations, it seems unlikely that most S-nitrosated sites could survive to stably regulate protein function. Consequently, disulfides would be the principal products that accumulate during nitrosative conditions. As predicted, disulfide formation was observed in candidate proteins, namely PKG1α, PKAR1, and PTEN, under conditions of nitrosative signaling induced by the transnitrosating NO donors CysNO, GSNO, or SNAP and the NO-releasing compound DETANO. Furthermore, disulfides accumulated prior to a detectable increase in S-nitrosation. Although protein S-nitrosation increased dose-dependently following CysNO treatment, this simply represents increased flux toward disulfide formation with enhanced levels of transient S-nitrosated intermediates, as would be anticipated. It would be erroneous to infer, as is most often the case, that these signals represent a global increase in stably S-nitrosated proteins. The transient nature of S-nitrosothiols and flux toward disulfides was further highlighted by examining the temporal changes in CysNO-induced S-nitrosation compared with disulfide formation. Indeed, CysNO induced an initial increase in S-nitrosation that then declined over time, whereas, under the same treatment, disulfides continued to accumulate. This is consistent with resolution of S-nitrosothiols to disulfides by reactions with abundant intracellular thiols.

Although it has previously been reported that PTEN phosphatase activity is inhibited by S-nitrosation (Numajiri et al., 2011), we find no evidence for this. After PTEN is exposed to CysNO, activity is only restored when the protein is reduced with DTT; this indicates that nitrosative signaling results in the formation of a regulatory disulfide that inhibits PTEN. The S-nitrosothiol reductant ascorbate failed to reduce oxidation of PTEN, whereas DTT, a disulfide reductant, efficiently achieved this. This further corroborates that the loss of reduced PTEN signal occurs because of a disulfide and not stable S-nitrosation. Peroxide-induced PTEN oxidation also likely occurs via a sulfenic acid intermediate that, like nitrosothiols, is a thiol-reducible intermediate (Zeller et al., 2009). In further support of disulfide-mediated functional regulation, calpain-1 was inhibited not only by CysNO but also by multiple thiol oxidants, including GSSSG and H_2_O_2_. This suggest that, although calpain-1 can be inhibited by nitrosating agents (Liu et al., 2016), this is likely via disulfide formation, not stable S-nitrosation.

As well as disulfide formation in candidate proteins, protein S-thiolation, specifically S-glutathiolation, was also significantly increased in cells exposed to CysNO or cell-permeable derivatives of GSNO. An inter-disulfide bond between a protein thiol and cysteine is termed S-cysteination. Treating cells with biotin-CysNO showed that widespread protein S-cysteination occurs, visualization of which is possible because of the biotinyl tag that otherwise cannot be detected using CysNO. This is consistent with an initial *trans*-nitrosation reaction to generate S-nitrosated proteins, followed by reductive removal of the NO by the action of the nascent cysteine to generate the disulfide. Protein S-cysteination in cells induced directly by CysNO itself, to our knowledge, has not been reported before.

Our observation that disulfides accumulate before detectable increases in protein S-nitrosation is supported by Wang et al. (2014), who showed that CysNO-induced S-nitrosation was accompanied by disulfide formation in candidate proteins such as DJ-1 and peroxiredoxin-2. Indeed, at a low concentration of CysNO (5 μM), they, like us, found that disulfides increased more than stably S-nitrosated protein. It was only when excess CysNO (>400 μM) was used that the amounts of S-nitrosated proteins surpassed those in the disulfide state. We hypothesize that this increase is due to a large bolus of NO donor-compromising cellular reducing systems, allowing the artificial stabilization of S-nitrosated proteins. Evidence supporting this comes from the finding that, although protein S-nitrosation accumulated at relatively low (15 μM) CysNO levels, these modifications were rapidly removed when a cell-permeable GSH analog was subsequently provided; the GSH analog resupplies the cells with thiol-reducing equivalents that rapidly react with the otherwise stabilized S-nitrosated proteins, removing them to generate disulfides. This emphasizes the instability of S-nitrosothiols and suggests that even “low” concentrations of CysNO, which may be a large amount for a cell monolayer, can compromise the intracellular reducing status. Furthermore, when cells were exposed to an even lower concentration of CysNO (10 μM), this significantly reduced the low-molecular-weight thiol pool and inhibited the NADPH-dependent reducing system. Consistent with this, when cellular GSH levels were depleted using buthionine sulfoximine or reduced by inhibition of the GSH-recycling enzyme GSNOR, CysNO-induced protein S-nitrosation was potentiated. Together, these findings indicate that widespread protein S-nitrosation occurs when the NADPH-thiol reducing system is compromised and that the use of exogenous NO donors is a significant factor in the detection of widespread S-nitrosation. When the cellular reducing systems were allowed to recover from a bolus of CysNO, protein S-nitrosation was no longer detected, whereas a significant amount of PKG1α disulfide was still observed. This again is consistent with S-nitrosothiols being labile, short-lived modifications under conditions where the cellular reducing system is intact and highlights the greater stability of disulfides, a feature that allows them to post-translationally regulate protein function and participate in homeostatic adaptation.

The evidence presented demonstrates that nitrosative signaling induces both widespread S-nitrosation and disulfide formation, with protein nitrosothiols transitioning to disulfide formation. However, the signals used to index protein S-nitrosation or disulfide formation are not directly or quantitatively comparable with each other because they use different analytical procedures. To compare quantitatively the level of S-nitrosation versus disulfide formation within individual samples, we performed a double S-nitrosothiol/disulfide switch analysis. This analysis confirmed again that exposure of cells to CysNO induced widespread cysteine thiol oxidation but demonstrated that disulfide was the predominant modification that accumulated. In addition, CysNO induced disulfides with a similar efficiency as the well-known thiol oxidants H_2_O_2_ and diamide. Thus, although CysNO induces widespread S-nitrosation that can be detected as a robust signal, this provides no information about the occupancy of the modification. We have to be careful not to interpret these as stoichiometric, regulatory post-translational modifications based on a robust banding pattern.

Although disulfides clearly quantitatively dominate over S-nitrosothiols after CysNO treatment, it is possible that there is a subset of proteins that are stably S-nitrosated and were missed because of the large and dominant disulfide signal. To address this, we coupled the double S-nitrosothiol/disulfide switch method with an unbiased, quantitative multiplexed proteomics approach involving iodoTMT labeling of cysteine thiols. This allows protein thiols to be quantitatively compared in terms of their occupancy as an S-nitrosothiol or a disulfide. Both CysNO and LPS, which stimulates endogenous NO production, increased S-nitrosation reproducibly, but the increase was minor compared with the amount of disulfides that concomitantly accumulated in the same samples. Almost every protein thiol that was found to be S-nitrosated was also present in the protein-S-disulfide state, and, importantly, the stoichiometry of the disulfide was greater than that of the S-nitrosothiol. The domination of protein disulfides compared with S-nitrosothiols after exogenous or endogenous nitrosative signaling is fully consistent with disulfides, not stable S-nitrosothiols, being the widespread end effectors in NO-mediated signaling. A heatmap highlights that the clear majority of individual, specific protein thiols was present in both the S-nitrosated as well as the disulfide state. That both oxidation states are present, with the disulfide quantitatively dominating in the vast majority of peptide thiols, provides further support for the transient nature of most S-nitrosothiols. To emphasize this crucial point, it was clear that treatment of cells with 10 μM CysNO caused a prominent and widespread protein S-nitrosation signal, as generated by the ascorbate-dependent biotin switch method. However, this robust signal can easily be misinterpreted but should not be construed as stoichiometric or as an end effector modification. This is because the occupancy of individual sites by NO is much lower than that for disulfides. Indeed, it is evident that disulfides accumulate to a much greater extent than anticipated from the chemistry highlighted here. Stable regulatory S-nitrosation does not occur to the extent many researchers in the field believe it to.

Given the vast number of publications on the subject, it is perhaps controversial and certainly thought-provoking to conclude that stable, regulatory S-nitrosation of proteins is unlikely to occur widely. Studies on protein S-nitrosation have escalated in recent times, perhaps because the primary method used (the ascorbate-dependent biotin switch method) can be readily applied and seemingly allows selective detection of protein S-nitrosation. The ability to index a modification and precisely define its chemical nature, especially when combined with cysteine mutagenesis studies, is considered a powerful approach. Thus, this method is valuable but perhaps a somewhat convenient choice. This method is typically used after a nitrosative intervention. Using an S-nitrosating agent in combination with a selective ascorbate-dependent method of nitrosothiol analysis helps focus attention on the modification of the protein by NO. This perhaps fosters a myopic view and draws attention away from the important role of S-nitrosation-dependent disulfide formation.

These data also highlight some of the complexity of directly comparing the effects of disparate nitrosating agents. For instance, although CysNO is cell-permeable and can rapidly and directly transnitrosate proteins (Singh et al., 1996), DETANO extracellularly releases authentic NO with a half-life of 20 hr (Keefer et al., 1996). Even when authentic NO enters the cell, it must first chemically transition to a reactive nitrogen species before it can participate in thiol oxidation reactions. With micromolar authentic NO thought to generate only nanomolar reactive nitrogen species, this reduces the ability of donors to induce protein S-nitrosation and subsequent nitrosothiol-dependent disulfide. This helps explains why donors of authentic NO are typically used at a higher concentration and for long durations to induce thiol modifications (Lim et al., 2008). However, the dominant product of both *trans*-nitrosating agents or authentic NO donors at nitrosating concentrations was disulfides, not stable S-nitrosothiols.

It is notable that there is significant contemporary literature advocating S-nitrosation as a *bona fide* post-translational modification that is selective, reversible, and enzymatically regulated. Such work can be biased, ignoring the inherent instability of nitrosothiols and their propensity to transition to more stable disulfides (Gladwin et al., 2002). The identification of so-called nitrosylases and denitrosylases that enzymatically regulate protein nitrosothiol levels is often presented as a keystone of regulatory S-nitrosation. A significant argument against the existence of such enzymes is that S-nitrosation reactions can occur rapidly and do not require enzyme catalysis. Furthermore, despite the Trx-TrxR system having a long and well-established role in reducing protein disulfides, it is increasingly described as a denitrosylase (Benhar et al., 2009). In such studies, it is typically assumed that Trx directly denitrosates proteins, and, therefore, when AF is used to prevent the regeneration of reduced Trx, increased S-nitrosation is observed. However, consistent with Trx as a disulfide reductase, AF will block the reductive removal of protein disulfides and indirectly limit a cell’s ability to remove S-nitrosated proteins because it also inhibits the nitrosothiol removal mechanism involving disulfide formation. Although Trx is expected to be capable of directly denitrosating proteins, as any thiol will likely react and remove the NO, this is not evidence that this event actually occurs in cells. It is likely that S-nitrosated proteins primarily first transition to disulfides before Trx reduces them. Although potentiation of CysNO-induced S-nitrosation has been reported (Shoman et al., 2011), it is notable, in our studies, that we found no evidence for this. We found that AF potentiated CysNO-induced disulfide bond formation in PKG1α. This suggests a limited role for Trx in direct protein denitrosation but is consistent with its long-established disulfide reduction role. Overall, we conclude that Trx as a global denitrosylase is questionable.

Inhibition of GSNOR also did not increase baseline S-nitrosation, calling into question its role as a denitrosylase. It is perhaps more likely that inhibition of GSNOR reduces the pool of GSH, stabilizing S-nitrosothiols after exposure to nitrosating agents. It is notable that cell-permeable GSH readily reversed protein S-nitrosation to generate S-glutathiolated proteins that are then reduced by glutaredoxin (Hill and Bhatnagar, 2012). Glutaredoxin, as a thiol-reactive enzyme, would be anticipated to directly reverse protein S-nitrosation; the question is whether it would compete with the rapid transition of such species to the S-glutathiolated state, which it will then also reverse. The same argument applies to Trx; in cells, does it primarily directly reduce S-nitrosated proteins or does it primarily do this after they first form disulfides?

Along with enzymatic regulation, another key feature of any post-translational modification is a consensus motif. Although initial studies provided evidence of a consensus motif, three other high-throughput proteomic studies failed to corroborate this (Feron et al., 1998, Gödecke et al., 1998, Hess et al., 1993). Even if there was a consensus motif for S-nitrosation, this might provide a basis for selective nitrosation-induced disulfide formation.

It is notable that many proteins long known to be regulated by disulfide formation are being “rediscovered” in the context of their regulation by S-nitrosation. As one example, cofilin is reported to be S-nitrosated at Cys80 (Zhang et al., 2015), whereas this residue is already known to form a disulfide (Klemke et al., 2008). In addition, some early examples of functional S-nitrosation, for example OxyR (Hausladen et al., 1996), have subsequently been found to be mediated by disulfides (Lee et al., 2004). A protein is typically shown to be S-nitrosated following a chemical nitrosative intervention with a coupled activity change. Generally, the stoichiometry of the modification is not determined but is assumed to be significant because there is a substantive change in activity. In some studies, the target cysteine of the reputed S-nitrosation has been mutated with the loss of activity or redox regulation. Mutagenesis of a target thiol will not only prevent its S-nitrosation but will also prevent the formation of the more stable, regulatory disulfide. Perhaps such oversights are caused by protein S-nitrosation being in vogue, although the complexities of selectively measuring global disulfide bond formation may also be influential.

Although S-nitrosothiols play an important role in cell signaling, the contemporary view of stable protein S-nitrosation as a widely occurring mechanism of post-translational regulation akin to phosphorylation is incorrect. Although protein S-nitrosation can be detected basally and increases during nitrosative interventions, evidence and arguments have been presented showing that this predominantly represents a short-lived modification that transitions to form a disulfide. We concede that some protein S-nitrosothiols are likely to be stable, especially when they are shielded from reducing thiols, making them resistant to reductive disulfide bond formation or stabilization by increased pH of the microenvironment (Paige et al., 2008). However, protein S-nitrosation as a ubiquitous regulatory mechanism is questionable, as is the evidence for a consensus motif that provides selectivity in NO targeting. Furthermore, Trx as a denitrosating protein can also be challenged; observations made using the TrxR inhibitor AF are open to several alternate interpretations. Other features of a post-translational regulatory mechanism include stoichiometric occupancy of the target amino acid. Although there are a few examples of this being achieved (Hamer et al., 2015), they have not been widely corroborated. With these considerations in mind, we suggest that the stoichiometry of this modification is considered before definitively concluding that a protein is directly regulated by stable S-nitrosation, as should the significant likelihood that it transitions to a disulfide that causatively mediates any associated functional alterations. Finally, it is notable that, instead of the chemically correct term of S-nitrosation, the term S-nitrosylation is often used. “Nitrosyl” chemically refers to NO bound to a transition metal, so apart from emphasizing parallels with phosphorylation, the inappropriate use of this term adds confusion.

## STAR★Methods

### Key Resources Table

**Table:** 

REAGENT or RESOURCE	SOURCE	IDENTIFIER
**Antibodies**		

Anti-glutathione	Virogen	Cat#101-A
Anti-PKA[R1]	BD Transduction Labs	RRID: AB_397566
Anti-PKG1α	Enzo Life Science	RRID: AB_2170659
Anti-α-Actin	Abcam	RRID: AB_2223021
PTEN Rabbit mAb	Cell Signaling	RRID: AB_390810
Streptavidin-HRP	Cell Signaling	RRID: AB_10830897

**Chemicals, Peptides, and Recombinant Proteins**		

lipopolysaccharide from *E. coli*	Sigma-Aldrich	Cat#L5024
recombinant human caplain-1	Abcam	Cat#ab91019
recombinant human PTEN	Enzo	Cat#BML-SE402-0010
buthionine sulfoximine	Sigma-Aldrich	Cat#B2515
N6022	Axon Medchem	Cat#21269
auranofin	Enzo	Cat#BML-EI206-0100
S-nitroso-N-acetyl-DL-penicillamin	Sigma-Aldrich	Cat#N3398
hydrogen peroxide	Sigma-Aldrich	Cat#H1009
Diamide	Sigma-Aldrich	Cat#D3648
1-nitrosocyclohexyl acetate	Sigma-Aldrich	Cat#SML0470
N-nitro-L-arginine methyl ester hydrochloride	Sigma-Aldrich	Cat#N5751
Diethylenetriamine/nitric oxide adduct	Sigma-Aldrich	Cat#D185
N-biotinyl-L-cysteine	Carbosynth	Cat#FB15460
Ez-Link NHS-Biotin	Thermo Fisher Scientific	Cat#20217
L-cysteine	Sigma-Aldrich	Cat#W326305
glutathione reduced ethyl ester	Sigma-Aldrich	Cat#G1404

**Critical Commercial Assays**		

calpain Activity Assay Kit	Abcam	Cat#ab65308
EnzChek Phosphatase Assay Kit	Thermo Fisher Scientific	Cat#E12020
Griess Reagent kit	Invitrogen	Cat#G7921

**Deposited Data**		

Raw and analyzed data	This paper; Mendeley Data	https://doi.org/10.17632/k2t8nx8kh5.1
Raw mass spectrometry dataset	Mendeley Data	https://doi.org/10.17632/k2t8nx8kh5.1
Swiss-Prot database with taxonomy set to Rattus (Oct 2016, 7,973 entries)	UniProt	http://www.uniprot.org/uniprot/?query=reviewed:yes%20taxonomy:10116

**Experimental Models: Cell Lines**		

Rat: primary vascular smooth muscle cells from aorta	Laboratory of Philip Eaton	N/A

**Software and Algorithms**		

Mascot search engine	Matrix Science	http://www.matrixscience.com/
Proteome Discoverer 1.4	Thermo Fisher Scientific	https://portal.thermo-brims.com/
R 3.2.5	RStudio 1.0.44	https://www.rstudio.com/
GelPro Analyzer 3.1	Media Cybernetics	http://www.mediacy.com/
Prism7	GraphPad	https://www.graphpad.com

### Contact for Reagent and Resource Sharing

Further information and requests for resources and reagents should be directed to and will be fulfilled by the Lead Contact, Philip Eaton (philip.eaton@kcl.ac.uk).

### Experimental Model and Subject Details

#### Materials

All laboratory chemicals were supplied by Sigma-Aldrich and tissue culture reagents by Life Technologies unless stated otherwise.

#### Cell culture

Primary vascular smooth muscle cells (SMCs) isolated from male Wustar rat aortas were cultured in DMEM GlutaMAX supplemented with 10% fetal bovine serum (FBS) and 1% penicillin/streptomycin. Cells were plated into 6, 12 or 96 well plates 48 hr before experiments. Experiments were performed on cells from passage 4 to 7.

All procedures were performed in accordance with the Home Office Guidance on the Operation of the Animals (Scientific Procedures) Act 1986 in the United Kingdom and were approved by the King’s College Animal Welfare and Ethical Review Body.

### Method Details

#### Induction of nitrosative or oxidative signaling in SMCs

The NO donor S-nitrosocysteine (CysNO) was generated by mixing 100 mM L-cysteine (Fluka Analytical) with 100 mM sodium nitrite in the presence of 100 mM hydrochloric acid (HCl) in the dark for 10 min. The reaction was then stopped by neutralisation with 100 mM sodium hydroxide. CysNO concentration was determined by measuring absorbance at 334 nm and using its extinction coefficient, 900 M^−1^ cm^−1^. A 100 mM stock was made up in Tris-HCl buffer pH 7.4 and pH checked to ensure it was within physiological limits. Cells were washed once with phosphate buffered saline (PBS) before treatment with CysNO (0-15 μM) in a final volume of 1 mL (6-well) or 0.5 mL (12-well) PBS for up to 30 min in the dark. Cells were washed in PBS after treatment with CysNO.

To compare cellular responses to nitrating agents to other oxidants, SMCs were treated for 5 min in PBS with vehicle control or 10 or 100 μM S-nitroso-N-acetyl-DL-penicillamin, 1-nitrosocyclohexyl acetate, H_2_O_2_ or diamide. Alternatively, SMCs were treated with 500 μM DETANO, an NO releasing compound, for up to 6 hr in media. After treatment all cells were washed with PBS before lysing into an appropriate buffer.

#### Detection of oxidative modifications in SMCs

##### S-nitrosation

S-nitrosation was detected in samples using the ascorbate-dependent biotin switch method whereby selectively removing SNO-modification and replacing it with a detectable biotin-tag. All steps were performed in the dark. Samples were harvested into lysis buffer (1% SDS, 100 mM maleimide, 0.2 mM neocuproine, 1 mM diethylene triamine pentaacetic acid, 100 mM Tris pH 7.4) and incubated at 50°C for 25 min with shaking to block free cysteine thiols. Samples were then desalted using Zeba Spin desalting columns (Thermo Fisher Scientific) into labeling buffer (0.5 mM biotin-maleimide, 0.5% SDS, 10 μM copper (II) sulfate, 60 mM ascorbate) and incubated in the dark for 1 hr at room temperature. Sample loading buffer (50 mM Tris-HCl pH 6.8, 2% SDS, 0.1% bromophenol blue, 10% glycerol) with the addition of 100 mM maleimide, was added to quench the labeling reaction. The samples were then resolved on a standard SDS-PAGE gel, immunoblotted, and probed with streptavidin-HRP (Cell Signaling). All immunoblots were analyzed with GelPro Analyzer 3.1.

##### Protein disulfides by immunoblotting

SMC lysates in sample buffer were resolved on a standard SDS-PAGE gel, immunoblotted, and probed with antibodies for PKAR1 (BD Transduction Labs) and PKG1α (Enzo Life Science). GelPro Analyzer 3.1 was used to quantify the proportion of disulfide dimer protein to reduced protein. To detect oxidation of PTEN, CysNO-treated SMCs were lysed into 1% SDS in 100 mM Tris pH 7.4. Samples were divided into three, incubated with 20 mM ascorbate, 20 mM DTT or vehicle and incubated for 30 min at 25°C with shaking. Sample loading buffer with the addition of 100 mM maleimide, was added to quench the reaction. Samples were then resolved on a standard SDS-PAGE gel, immunoblotted, and probed with an antibody for PTEN (Cell Signaling). The proportion of reduced to oxidised PTEN was then quantified.

##### S-glutathiolation

SMCs treated with 0-10 μM CysNO were harvested into sample buffer with the addition of 100 mM maleimide. The samples were then resolved on a standard SDS-PAGE gel, immunoblotted, and probed with an anti-glutathione antibody (Virogen) to detect protein S-glutathiolation.

##### N-biotinyl S-cysteination

S-cysteination resulting from treating SMCs with CysNO was detected using biotin-labeled cysteine. N-biotinyl-L-cysteine (BioCys, Carbosynth) was used in place of L-cysteine to synthesize CysNO resulting in the formation of BioCysNO. 100 mM BioCys was reacted with 100 mM hydrochloric acid in the dark for 10 min followed by neutralisation with 100 mM sodium hydroxide as a control. SMCs were treated with 10 μM BioCysNO or BioCys for 30 min in the dark. Cells were washed twice with PBS and harvested into sample buffer with the addition of 100 mM maleimide. Samples were then immunoblotted with Streptavidin-HRP to detect N-biotinyl S-cysteination.

##### N-biotinyl S-glutathiolation

S-glutathiolation resulting from treatment of SMCs with the NO donor S-nitrosoglutathione (GSNO) was detected using cell-permeable biotin-labeled glutathione (BioGSH). N-biotinyl GSH ethyl ester (BioGEE) was synthesized by reacting equimolar GSH ethyl ester (GEE)with Ez-Link NHS-Biotin (Thermo Fisher Scientific) in the presence of 0.6 mM diethylene triamine pentaacetic acid at 42°C for 30 min. The reaction was quenched with the addition of 100 mM Tris-HCl. N-biotinyl S-nitrosoglutathione ethyl ester (BioGSNOEE) was synthesized by reacting equimolar BGEE and sodium nitrite in the presence of 100 mM hydrochloric acid for 10 min in the dark. S-nitrosoglutathione ethyl ester (GSNOEE) was synthesized by reacting equimolar GEE and sodium nitrite in the presence of 100 mM HCl for 10 min in the dark. GSNO was synthesized by reacting equimolar GSH with sodium nitrite in the presence of 100 mM HCl for 10 min in the dark. The concentrations of BioGSNOEE, GSNOEE and GSNO were determined by measuring absorbance at 345 nm and using an extinction coefficient of 920 M^−1^ cm^−1^. SMCs were treated with 0-10 μM BioGEE, BioGSNOEE, GSNOEE or GSNO for 30 min in a final volume of 1 mL of PBS in the dark. Cells were washed twice with PBS and harvested into SDS sample buffer supplemented with 100 mM maleimide. Samples were then immunoblotted and probed with Streptavidin-HRP to detect N-biotinyl S-glutathiolation.

##### Double S-nitrosothiol/disulfide switch

A modified version of the ascorbate-dependent biotin switch was used to detect total disulfides or S-nitrosothiols + disulfides, hereby referred to as the double S-nitrosothiol/disulfide switch and based on the d-SSwitch method described by Wang et al. (2014). Samples were harvested into lysis buffer and blocked. Samples were then spilt into three – non-reduced control, disulfides and S-nitrosothiols + disulfides. The “Disulfides” sample was desalted into ascorbate reducing buffer (100 mM maleimide, 0.5% SDS, 10 μM copper (II) sulfate, 60 mM ascorbate); “control” and “S-nitrosothiols + disulfide” samples were desalted into 1% SDS. All samples were then incubated at room temperature for 1 hr. “Control” sample was desalted again into 1% SDS while “disulfide” and “S-nitrosothiols + disulfides” samples were desalted into reducing buffer containing 20 μM tris(2-carboxyethyl)phosphine and incubated for 10 min at 60°C. Finally, all samples were desalted into labeling buffer (0.5 mM biotin-maleimide, 0.5% SDS) and incubated at room temperature for 1 hr before the reaction was quenched with the addition of sample loading buffer containing 100 mM maleimide. The samples were resolved on a standard SDS-PAGE gel, immunoblotted, and probed with streptavidin-HRP. GelPro Analyzer 3.1 was used to quantify oxidative modification of proteins.

#### Protein activity assays

##### PTEN activity assay

The enzymatic activity of PTEN was measured using an EnzChek Phosphatase Assay Kit (Thermo Fisher Scientific). In brief, recombinant human PTEN (Enzo) was reconstituted in a buffer (150 mM sodium chloride, 2 mM disodium ethylenediaminetetraacetate dehydrate [EDTA.Na_2_], 50 mM Tris-HCl pH 7.4) at a concentration of 2.2 μM, supplemented with 25 μM DTT and incubated at room temperature for 10 min to reduce the protein. The sample was then divided in two and exposed to vehicle or 200 μM CysNO for 10 min at room temperature in the dark. The samples were then desalted using Zeba Spin desalting columns (Thermo Fisher Scientific) into degassed reaction buffer (2 mM EDTA.Na_2_, 0.05% Triton X-100, 100 mM Tris-HCl pH 8) and plated into 96-well plate with PTEN at a final concentration of 200 nM. A 20-fold excess of S-nitrosothiol specific reductant sodium-L-ascorbate or disulfide reductant DTT was added to the samples and incubated for 5 min at room temperature in the dark. The activity assay was initiated with the addition of 200 μM 6,8-difluoro-4-methylumbelliferyl phosphate (DiFMUP). Fluorescence was measured at Ex 360 nm/ Em 450 nm at 1 min intervals for 60 min using a SpectaMAX GEMINI XS fluorometer and the data were analyzed with SOFTmax Pro (v. 4.51). The amount of product produced was calculated against a standard curve, with 0-10 μM 6,8-Difluoro-7-hydroxy-4-methylcoumarin as reference standards.

##### Calpain activity assay

The enzymatic activity of calpain-1 was measured using Calpain Activity Assay Kit (Abcam). In brief, 1.53 μg recombinant caplain-1 (Abcam) per reaction was desalted into reaction buffer (100 mM Tris pH 7.4 containing 50 mM CaCl_2_) using Zeba Spin desalting columns and reacted with 10 μM CysNO, disulfide glutathione, diamide, H_2_O_2_ or the irreversible calpain inhibitor Z-LLY-FMK for 30 min at 37°C. A fluorescent calpain substrate was added to each sample and fluorescence at Ex 400 nm/ Em 505 nm was measured at 5 min intervals for 60 min.

##### NAPDH-dependent enzyme activity

The activity of NADPH-dependent enzymes was measured using CellTiter 96 AQueous One Solution Reagent, which contains a tetrazolium dye [3-(4,5-dimethylthiazol-2-yl)-5-(3-carboxymethoxyphenyl)-2-(4-sulfophenyl)-2H-tetrazolium, inner salt; MTS] and an electron coupling reagent (phenazine ethosulfate). Reduction of MTS by NADPH-dependent enzymes produces a colored product that is measured spectrophotometrically. SMCs were grown in a 96-well plate and then treated with 0-10 μM CysNO in PBS in the dark at 37°C. After incubation, cells were washed once with PBS and then left to recover in 100 μL media for 0 or 60 min before addition of 20 μL CellTiter 96 AQueous One Solution Reagent. Cells were incubated in the dark for 1 hr at 37°C and the absorbance measured at 450 nm.

#### Measuring non-protein and total thiols

A standard Ellman’s assay was used to determine non-protein and total thiols in samples. To measure total thiol 20 μL of the sample was incubated in buffer containing 30 mM Tris-HCl pH 8.2, 3 mM ethylenediaminetetraacetic acid (EDTA) and 150 μM 5,5′-dithiobis(2-nitrobenzoic acid) (DTNB) made up to a final volume of 520 μL with methanol. To assay for non-protein thiols 50 μL of sample was incubated in 10% trichloroacetic acid on ice for 15 min and then protein pelleted at 15,000 x g for 5 min before isolating the supernatant. Ellman’s assay for non-protein thiols contained 50 μL of supernatant with the addition of 30 mM Tris-HCl pH 8.9, 3 mM EDTA and 150 μM DTNB. Assay mixtures were spun down at 3,000 x g for 5 min and absorbance measured at 450 nm. Thiol content was determined by comparison to a standard curve produced by assaying GSH (0-1000 μM).

#### Inhibition of GSH synthase in SMCs

SMCs were treated with or without 100 μM buthionine sulfoximine for 3 days in complete media. Following this they were washed with PBS and treated with or without 5 μM CysNO for 30 min before washing with PBS. Cells were harvested in lysis buffer before undergoing the ascorbate-dependent biotin switch and immunoblotting to detect level of S-nitrosation.

#### Inhibition of S-nitrosoglutathione reductase in SMCs

SMCs were treated with or without 25 μM N6022 (Axon Medchem) for 1 hr in complete media to inhibit S-nitrosoglutathione reductase. Following this cells were washed with PBS and treated with or without 5 μM CysNO for 30 min before washing with PBS. Cells were harvested in lysis buffer before undergoing the ascorbate-dependent biotin switch and immunoblotting to detect level of S-nitrosation.

#### SMCs treatment with NO donor and glutathione

Cells were treated with 15 μM CysNO for 30 min in the dark, washed once with PBS then treated with 0-1 mM GSH ethyl ester for 30 min in the dark. Cells were then washed with PBS, harvested in lysis buffer and analyzed using the ascorbate-dependent biotin switch as described above to detect the level of S-nitrosation.

#### Inhibition of thioredoxin reductase in SMCs

SMCs were treated with or without 2 μM auranofin (Alexis), a gold complex known to inhibit thioredoxin reductase, for 1 hr in media without FBS at 37°C. Cells were then washed once with PBS before treatment with or without 15 μM CysNO for 30 min in the dark at 37°C in PBS. After incubation cells were washed twice with PBS, lysed and then analyzed using the ascorbate-dependent biotin switch and western blotting.

#### Measuring nitrite production in SMCs

SMCs were treated with 1.5 μg/mL lipopolysaccharide (LPS) from *E. coli* for 18 hr to stimulate endogenous NO production. The Griess assay was preformed using a kit (Invitrogen) to measure the production of nitrite. In brief, after LPS stimulation, conditioned media was collected and added to Griess reagent [1% (w/v) sulfanilamide and 0.1% (w/v) N-(1-naphthyl) ethylenediamide in 5% (v/v) phosphoric acid]. After 30 min incubation in the dark, absorbance was measured at 570 nm and the nitrite concentration was calculated using a series of sodium nitrite dilutions to generate a nitrite standard curve.

#### IodoTMT mass spectrometry

##### IodoTMT switch labeling of S-nitrosothiols and disulfides in SMCs

S-nitrosothiols and disulfides in SMCs were labeled using the iodoTMTsixplex Isobaric Mass Tagging Kit (Thermo Fisher Scientific). IodoTMT labeling was conducted in the dark using a modified double S-nitrosothiol/disulfide switch method. SMCs treated with vehicle, 10 μM CysNO for 30 min or 1.5 μg/mL LPS for 18 hr were lysed in iodoTMT lysis buffer (1% SDS, 20 mM iodoacetamide, 0.2 mM neocuproine, 1 mM diethylene triamine pentaacetic acid, 100 mM Tris-HCl pH 7.4) and incubated at 50°C for 30 min to block thiol groups. A standard bicinchoninic acid assay was performed and samples diluted to a protein concentration of 1.5 mg/mL before proceeding with labeling. To remove excess iodoacetamide, samples were desalted using 5 mL Zeba Spin desalting columns (Thermo Fisher Scientific) into labeling buffer (0.5% SDS, 100 mM Tris pH 7.4). To label S-nitrosothiol thiols, 20 mM ascorbate and 0.4 mM iodoTMT reagent was added and samples were incubated for 1 hr at room temperature. Samples were then desalted into labeling buffer with the addition of 20 mM Bond-Breaker tris(2-carboxyethyl)phosphine (TCEP, Thermo Fisher Scientific) and incubated at 60°C for 10 min to break disulfides. Samples were desalted into labeling buffer with the addition of 0.4 mM iodoTMT reagent and incubated for 1 hr at room temperature to label disulfides. The reaction was quenched with the addition of 15 mM dithiothreitol with incubation for 15 min at 37°C. The three samples (control, CysNO-treated or LPS-treated) individually labeled by iodoTMTsimplex reagents were then combined before tryptic digestion.

##### In-solution tryptic digestion

Protein was precipitated before digestion with the addition of 6 volumes of cold acetone and incubated at −20°C overnight. Samples were then centrifuged at 10,000 x g for 10 min at 4°C. The pellet was air-dried for 10 min and resuspended in 50 mM ammonium bicarbonate pH 8 containing 20 μg/mL trypsin and digested overnight at 37°C. The sample was centrifuged at 2,500 x g for 10 min and the supernatant was lyophilized using a vacuum concentrator.

##### Sample fractionation

Samples were resuspended in 2% phosphoric acid and desalted using Oasis HLB 1CC cartridges (Waters) according to the manufacturer’s instructions. Vacuum-dried samples were resuspended in HPLC grade water with 20 mM ammonium formate for high-pH reversed phase fractionation on an Agilent 1100 series HPLC with a Poroshell 300 Extended C18 column (Agilent). Fractions were collected from 1-37 min at 1.2 min intervals at a flow rate of 200 μL/min using a gradient of 100% acetonitrile + 20 mM ammonium formate. Collected fractions were vacuum dried, resuspended in water and vacuum dried again.

##### Mass Spectrometry

Individual fractions were analyzed by nanoLC-MS/MS with an Ultimate 3000 LC system coupled to an LTQ Orbitrap XL instrument (Thermo Fisher Scientific). Samples were injected and peptides separated by reversed phase nano LC (Acclaim Pepmap 100Å 75 μm x 25 cm column, Thermo Fisher Scientific) with an X5 Pepmap pre-column over a 90 min gradient of 10%–50% acetonitrile + 0.1% formic acid at a flow rate of 300 nl/min. The column was washed with 100% acetonitrile/formic acid for 10 min before equilibrating with 10% acetonitrile/formic acid for 15 min. Data-dependent acquisition was performed on the top 3 most intense ions, with CID and HCD performed in the ion trap and Orbitrap, respectively. Dynamic exclusion was enabled with a list size of 500 for 15 s and only ions with at least a +2 charge were targeted for MS/MS.

### Quantification and Statistical Analysis

#### Immunoblot and activity assay analysis

All immunoblot bands were quantified using GelPro Analyzer 3.1 (Media Cybernetics). All datasets were performed with a n ≥ 3 and are presented as mean ± the standard error of the mean (SEM). Differences between groups were assessed using ANOVA where appropriate, followed by appropriate post hoc analysis using Prism7 (GraphPad). Differences were considered significant at the 95% confidence level (p < 0.05).

#### Mass spectrometry data searching and analysis

Mass spectrometry raw data files were searched using the Mascot search engine (Matrix Science) with Proteome Discoverer 1.4 (Thermo Fisher Scientific). CID and HCD data were searched against the Swiss-Prot database with taxonomy set to Rattus (Oct 2016, 7,973 entries). N terminus acetylation, NQ deamidation of, M oxidation, carbamidomethylated C and iodoTMTsixplex C were set as dynamic variables. Tandem mass tag intensities and ratios were calculated by the software without normalization and using the minimum intensity for missing tags. Differences between groups for mass spectrometry data were assessed using a Mann-Whitney U test. Peak areas were also reported and the individual tag intensities used to deconvolute peak areas for individual peptides by multiplying the peak area by the ratio of individual tag intensity to the sum of all tag intensities for each peptide. Peptide peak area values and analysis was performed in R 3.2.5 in R Studio 1.0.44.

### Data and Software Availability

Raw mass spectrometry dataset is deposited on Mendeley Data (https://doi.org/10.17632/k2t8nx8kh5.1).
